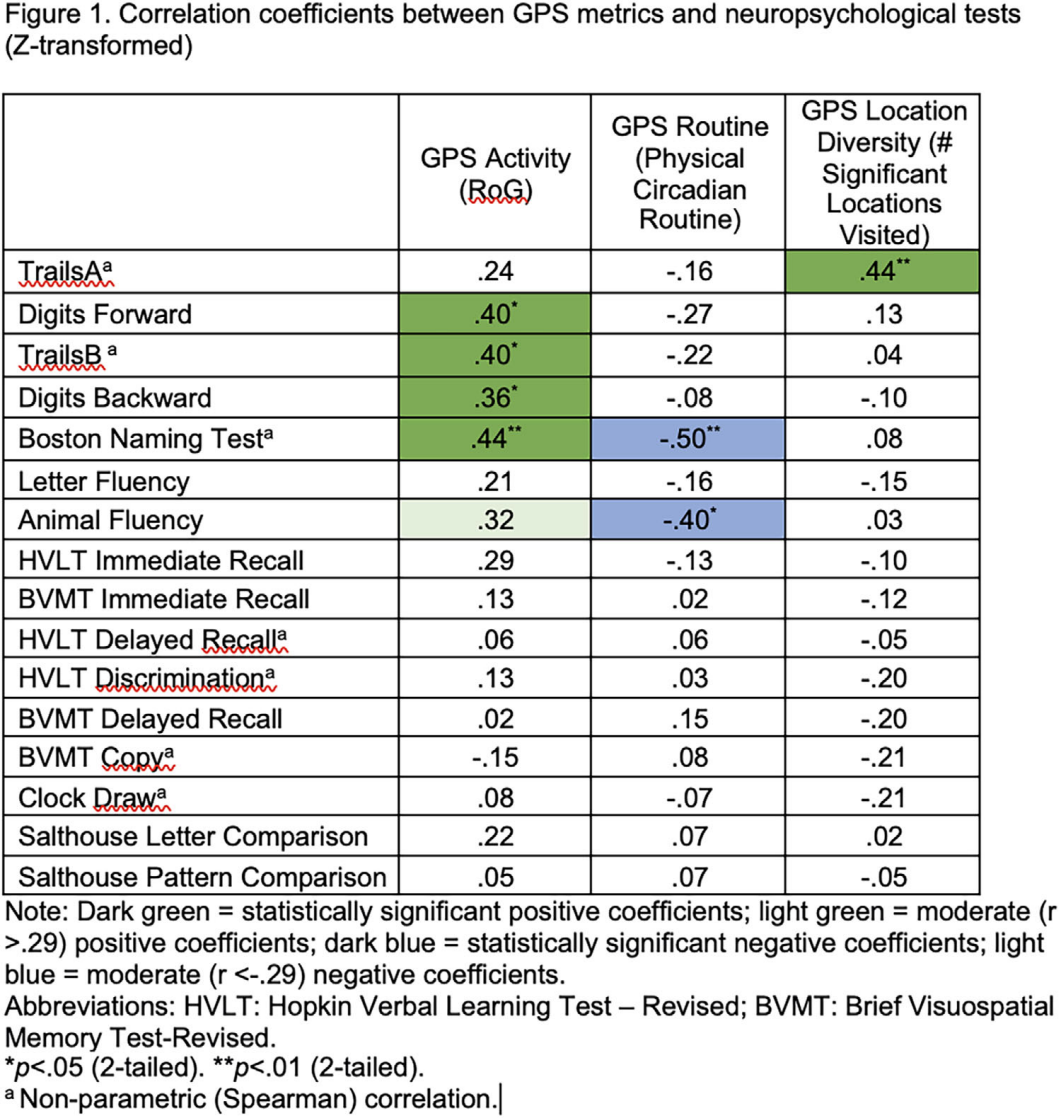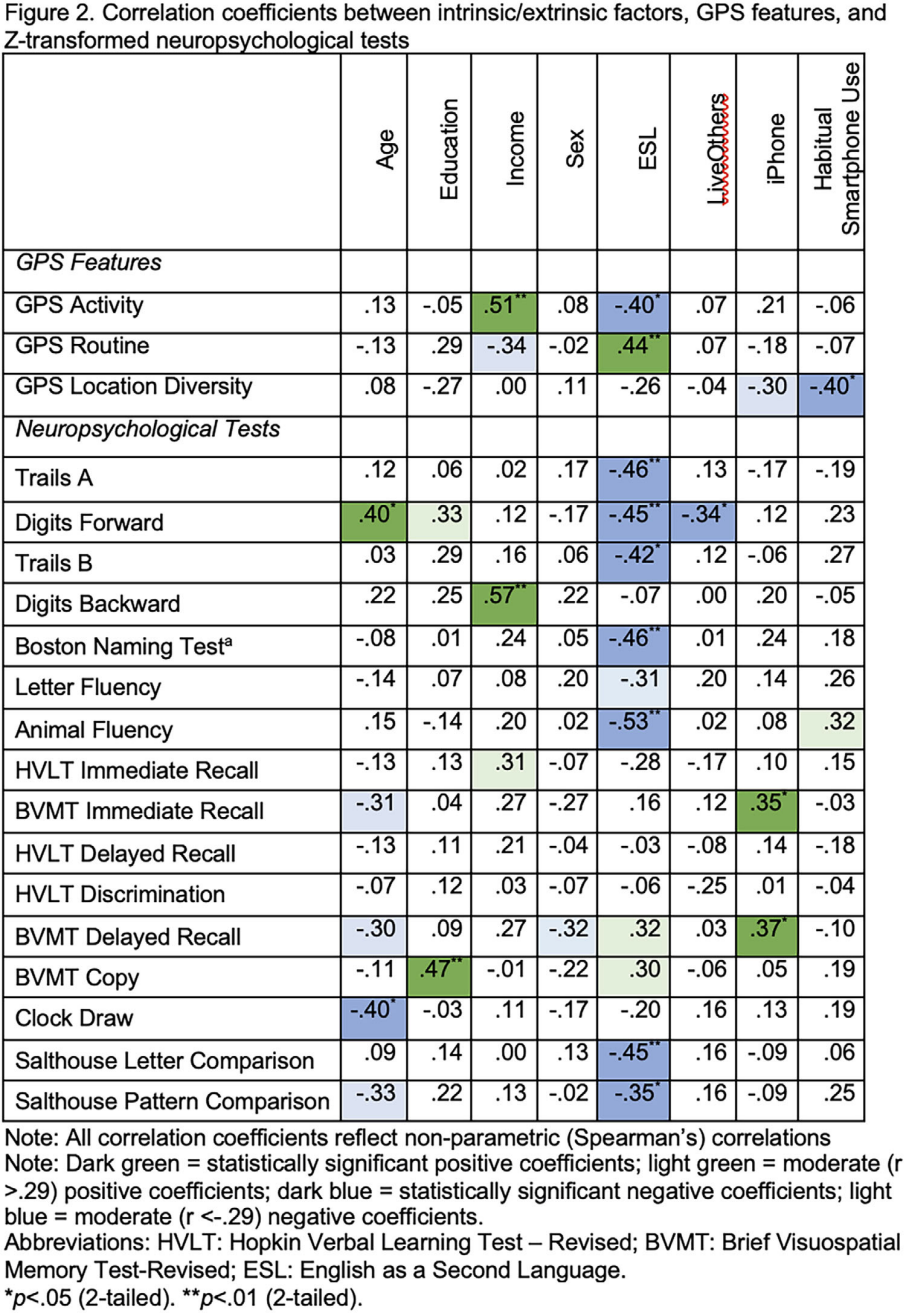# Exploring the differential impact of sociodemographic and extrinsic factors on mobility‐based digital phenotypes versus standard cognitive tests among older adults

**DOI:** 10.1002/alz.093355

**Published:** 2025-01-03

**Authors:** Katherine Hackett, Shiyun Xu, Moira McKniff, Ian Barnett, Tania Giovannetti

**Affiliations:** ^1^ Icahn School of Medicine at Mount Sinai, New York, NY USA; ^2^ University of Pennsylvania Perelman School of Medicine, Philadelphia, PA USA; ^3^ Temple University, Philadelphia, PA USA

## Abstract

**Background:**

Passively‐obtained smartphone digital phenotypes may yield objective estimates of everyday cognition in older adults compared to traditional cognitive/self‐report measures typically confounded by sociodemographics. However, it is currently unknown what covariates are relevant when interpreting smartphone sensor data. We aimed to clarify which intrinsic and extrinsic factors are associated with digital phenotyping versus traditional cognitive measures in a cohort of older adults.

**Methods:**

34 participants (M_age_ = 71.6±5.5; M_education_ = 16.4±2.7; 57% non‐Hispanic White; 88% native English language) with normal cognition or Mild Cognitive Impairment used an open‐source smartphone application (mindLAMP) to passively capture GPS trajectories for one month. Baseline neuropsychological tests were collected as external validators along with self‐reported sociodemographics (intrinsic) and technology habits/phone type (extrinsic). GPS data were processed into monthly feature estimates of activity, routine, and location diversity. Correlations and univariate ANOVAs were used to examine relationships between GPS features, z‐transformed neuropsychological tests, and intrinsic/extrinsic factors.

**Results:**

Consistent with prior findings, greater monthly GPS activity (radius of gyration), less routine (physical circadian routine), and more location diversity (significant locations visited) associated with higher scores on measures of attention, executive functioning, and language (.36≤|*r’s*|≤.50, *p*’s<.05). Several sociodemographic factors associated with cognitive scores but not with GPS features; these included age, education, living alone (.34≤|*r’s*|≤.47, *p*’s<.05) and race (3.8≤F(2,30)≤ 5.5, .20<η^2^<.27, *p’s*<.05). Higher lifetime income and native English language associated with better cognitive scores and with more GPS activity/less routine (.35≤| *r’s*|≤.57, *p*’s<.05). Sex and current occupational status were not associated with cognitive tests or GPS features. iPhone vs. Android ownership associated with cognitive tests but not GPS metrics (.35≤*r’s*≤.37, *p*’s<.05), whereas more habitual smartphone use associated with less location diversity but not with other GPS metrics or cognitive tests (*r = ‐*.40, *p*<.05).

**Conclusions:**

Passively‐obtained mobility phenotypes were significantly and moderately correlated with gold standard neuropsychological tests commonly used for clinical assessment and clinical trial endpoints. Several factors pertinent to social determinants of health were related to neuropsychological tests but not GPS features, suggesting naturalistic assessment via digital phenotyping may be less biased by these factors. On the other hand, other important factors including lifetime income and native English language impacted both methods of assessment.